# Bilateral Simultaneous Temporal Relapses of Medulloblastoma

**DOI:** 10.7759/cureus.2170

**Published:** 2018-02-08

**Authors:** Mauricio Martinez-Moreno, Olga O Galván de la Cruz, Christian H Flores-Balcázar, Samuel Rosales-Pérez, Daniel Rembao-Bojórquez, Sergio Moreno-Jiménez

**Affiliations:** 1 Neurosurgery, HELIOS Ostseeklinik Damp; 2 Radioneurosurgery Unit, National Institute of Neurology and Neurosurgery; 3 Department of Radiation Oncology, National Institute of Nutrition and Medical Sciences; 4 Radiation Oncology Department, Oncology Hospital, Cmn Siglo Xxi; 5 Neuropathology Department, National Institute of Neurology and Neurosurgery; 6 Neurosurgery-RadioSurgery, American British Cowdray Medical Center

**Keywords:** medulloblastoma, bilateral relapse, supratentorial

## Abstract

Supratentorial relapses are a common component of medulloblastoma after failure of treatment. Craniospinal irradiation (CSI) to cerebrospinal fluid-bearing areas is an essential part of the management of these tumors both in adults and children. Failure of treatment in specific anatomical regions can be attributable to technical inaccuracies in CSI technique leading to radiation underdosing in such areas. We present two cases of patients with bilateral simultaneous metastasis of a primary medulloblastoma treated, in both cases, four years before the recurrence. In both patients the tumors were mirror images, at the right and left temporal pole. Radiotherapeutic plans were analyzed in both cases, and a possible mechanism determining the pattern of relapse is discussed. We consider, in agreement with the literature, that a prone position during treatment, shielding blocks at the cribiform/subfrontal region, and anatomic inadequacies in the CSI fields could have contributed to the presented pattern of relapse.

## Introduction

Medulloblastoma is a malignant neuroectodermal tumor with a high potential for distant tumor dissemination. Although uncommon in the adult population, it accounts for approximately 30% of all brain tumors of childhood. In the adult population medulloblastoma represents around 3% of all primary neoplasms of the central nervous system. The use of postoperative craniospinal radiotherapy is the single most important reason for the improvement over the last four decades in patients' five-year survival rate [1]. The conventional doses are 36 Gy to the spinal axis and 54 Gy to the posterior fossa. Careful radiation therapy technique includes adequate irradiation of the neuroaxis, with special attention to the cribiform plate region and the termination of the thecal sac. Leptomeningeal treatment failure commonly occurs in the leptomeninges of the posterior fossa and spine. The posterior fossa is the most common site for isolated tumor recurrence; however, relapses in the supratentorial region, especially in the cribiform plate subfrontal area, are not rare [2].

## Case presentation

We report two cases with bilateral, simultaneous temporal recurrences. 

Case 1

**Figure 1:**
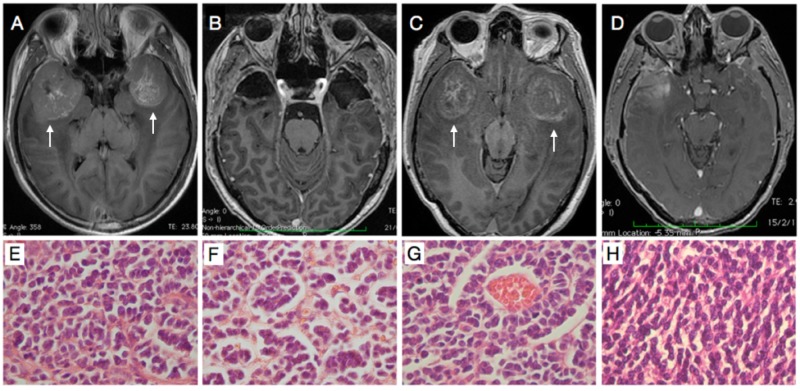
Radiologic and histopathologic representative images Radiologic (A-D) and histopathologic (E-H) representative images of the presented cases. An MRI after gadolinium injection showed a 5 cm globular mass with heterogeneous enhancement and peripheral edema over both temporopolar areas in Case 1 (white arrows)(A). The postoperative MRI after gadolinium injection demonstrated total gross resection in the patient after two surgical procedures (B). The histopathology report confirmed a classic medulloblastoma for both tumors as microscopically visualized with H/E staining technique (E and F; right and left tumor, respectively; Hematoxilin/Eosin, 40x,). In Case 2, a T1-weighted magnetic resonance imaging after injection of gadolinium similarly showed a mass with heterogeneous enhancement and peripheral edema on both temporal poles (white arrows) (C). After surgical management, a postoperative MRI with contrast medium demonstrated total gross resection of both tumors (D). The neuropathology report confirmed classic medulloblastoma in the right and left tumors of Case 2 (G and H, respectively; Hematoxilin/Eosin 40x). MRI - Magnetic resonance imaging.

A 34-year-old male presented to our service four years after surgery in another hospital for a posterior fossa tumor reported as a medulloblastoma. The posterior fossa tumor was excised, and gross total resection was achieved at that time. Pathology reported the tumor to be consistent with medulloblastoma. Spinal magnetic resonance imaging (MRI) and a lumbar puncture did not demonstrate neuroaxis dissemination. The patient received standard craniospinal irradiation (CSI) consisting of 36 Gy, a boosted primary posterior fossa site dose of 54 Gy, in prone position, and chemotherapy. When he presented at our hospital with a two-week history of headache, somnolence, nausea, and vomiting; his family reported hypersexuality and abulia during the last four months. The MRI demonstrated two tumors located at each middle cranial fossa in the temporal poles (Figure 1A).

He was admitted with a presumptive diagnosis of medulloblastoma seedings. The posterior fossa and spine were without evidence of tumor. Both tumors were surgically treated at different times (Figure 1B). Macroscopically, both tumors comprised soft purplish tissue easily cleavable from the surrounding brain parenchyma and moderate vascularization. The histopathological diagnosis was classic medulloblastoma for both tumors (Figure 1E-1F).

Case 2

A 38-year-old male with a history of hydrocephalus secondary to a tumor located in the posterior fossa had been treated six years before in another hospital. At that time, the patient underwent ventriculo-peritoneal shunt and tumor resection. The histopathological report was medulloblastoma. The patient received standard craniospinal irradiation (CSI) consisting of 36 Gy, a boosted primary posterior fossa site dose of 54 Gy, in prone position, and chemotherapy. Four years after the surgery, he presented with progressive memory impairment and abulia. Finally, he presented with somnolence, which was the reason why he was brought to our emergency department. The patient underwent an MRI where two well-circumscribed lesions, in both middle floors and no tumoral activity in the posterior fossa were demonstrated (Figure 1C). Both tumors were resected in separate surgeries achieving gross tumor total resection on either side (Figure 1D). The histopathology also demonstrated a classic medulloblastoma in both right and left tumors (Figure 1G-1H, respectively).

## Discussion

Late tumor relapse is common among adult patients with medulloblastoma; thus, long-term follow-up is essential when monitoring these patients. Leptomeningeal treatment failure is a common component of treatment failure occurring mainly in the leptomeninges from the posterior fossa and spine. According to the literature, the posterior fossa is the most frequent site of isolated tumor relapse in adults; however, relapses in supratentorial areas are not rare [3].

CSI is an essential component of the adjuvant therapy for medulloblastoma, and CSI technique for such tumors involves irradiation of all cerebrospinal fluid (CSF)-bearing areas. These areas are at risk for tumor seeding because these tumors mainly disseminate via the CFS. Inadequacies in CSI technique may lead to underdosing with radiation and subsequently produce “cold spots”, which have the potential of serving as a nidus for tumor recurrence.

In 1987, Miralbell et al. analyzed the factors that potentially influence the site of failure in pediatric medulloblastomas and found a correlation between a correct field of whole brain irradiation and supratentorial failure-free survival. The authors suggested that treatment protocols that limit supratentorial irradiation to subsites at the highest risk of relapse should be considered. They presented a case with a subfrontal relapse arising from the shielded cribiform region [4].

In the same year, Hartsell et al. evaluated pre-irradiation chemotherapy on patterns of failure in children with medulloblastoma and reported more relapses in the posterior fossa than in the neuroaxis, 11 and four, respectively. Following CSI, neuroaxis progression was more frequent than posterior fossa relapse [5].

A multicenter retrospective study of 72 adolescents aged 10 to 20 years with medulloblastoma was reviewed. Late relapses occurred at a median of three years (range 0.3–6.8 years) in 20 patients. The local posterior fossa with and without leptomeningeal dissemination was the most common site of relapse. In the aforementioned study, the patients were classified regarding relapse location as local, distant, or combined. Although a lower dose of CSI was associated with a higher rate of distant relapses, the numbers were too small to reach statistical significance [6].

Chan et al. analyzed patterns of relapse and prognostic factors for adult patients with medulloblastomas (aged 16–47 years). The median time to recurrence was 26 months, with a range of four to 109 months. In our study, the first patient presented with the initial recurrence after 48 months. Our second patient relapsed initially after 72 months, and the second time 48 months later. In the series of Chan et al., only 29% of all relapses occurred more than five years after treatment. The posterior fossa was the most common site of relapse, and the authors reported only five out of 32 relapses in the supratentorial space without specifying the site [7].

The CSF flows in the subfrontal/cribiform plate region. When a skull is viewed from above, this region lies between the orbital roofs. When viewed laterally, one can readily see that the orbital roof is above the cribiform region. Thus, a lateral block for the whole-brain irradiation field designed to spare the eyes may block out the cribiform plate region. This has the potential to provide a nidus for tumor relapse as stated by the authors [3].

**Figure 2:**
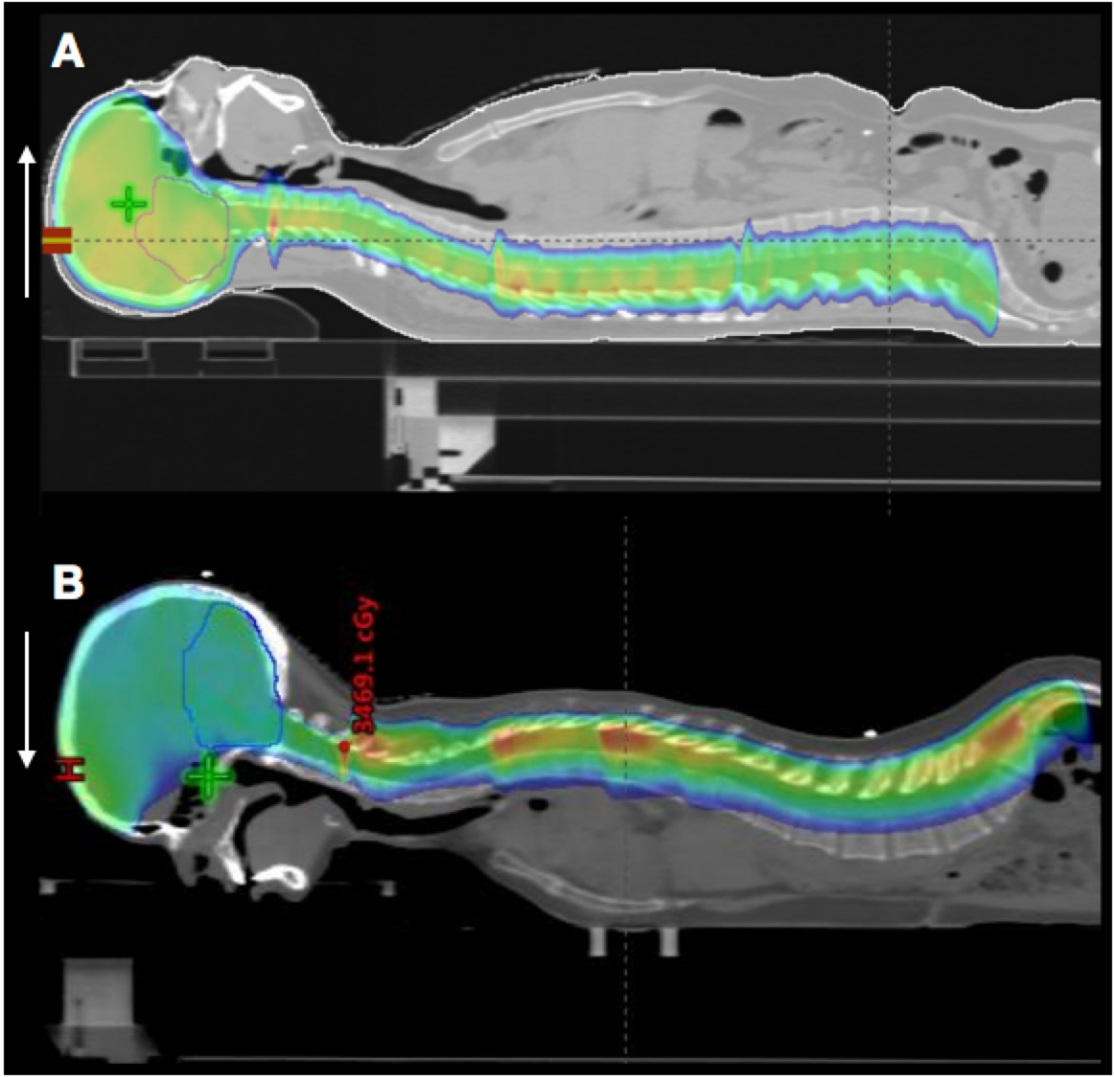
Prone vs supine position for radiotherapy treatment Radiotherapeutic plan for CSI in supine position (white arrow) (A). Radiotherapeutic plan for CSI in prone position; protection blockage of the eyes could contribute to underdosing of the temporopolar area located just behind the created shield. A prone position during the CSI session could also contribute in a gravity-related fashion to tumor seeding (white arrow) (B). CSI - Craniospinal irradiation.

According to Halprin et al. cribiform-subfrontal recurrences possibly result from the prone position of the patient during surgery or radiotherapy procedures (Figure 2) [8].

In such a position, medulloblastoma cells float in a gravity-dependent fashion to the cribiform plate region, consequently favoring malignant cells seeding and tumor relapse. It is also possible that an excessively generous lateral brain block used to shield the eyes during radiation allows tumor to recur in the cribiform region. Of 22 identified relapses, 10 were at least partially attributable to failure of CSI technique [8].

More recently, Taylor et al. analyzed the impact of targeting deviations during CSI on tumor relapse. They found a significant impact of posterior fossa targeting deviations on posterior fossa recurrences. According to their data, supratentorial relapses were not significantly related to targeting deviations of the skull base or cribiform target volumes [9].

Ramaswamy et al. recently published the largest series of recurrent medulloblastomas in patients who were mostly aged below 16 years. In contrast to the literature, they found distant metastasis as the most common pattern of tumor relapse compared with local relapse; however, the authors focused on the relationship between recurrence patterns and specific molecular groups (Groups 3 and 4) of medulloblastoma [10].

## Conclusions

In conclusion, the CSI protocol should take care to avoid underdosing at the junction of the cranial and spinal fields, and at the sub-frontal and temporal areas. A supine position is a good option.
